# Raymond Schinazi Discusses the Discovery of Early Antiretroviral Agents and Sofosbuvir

**DOI:** 10.20411/pai.v8i1.624

**Published:** 2023-11-06

**Authors:** Michael M. Lederman, Neil S. Greenspan

**Affiliations:** 1 Case Western Reserve University, Cleveland, Ohio

*This interview has been edited for clarity.

**Figure:**
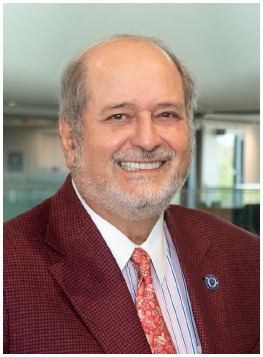
Raymond Schinazi, PhD, Hon DSc

In this interview, Raymond Schinazi, PhD, Hon DSc, talks about his career with Michael M. Lederman, MD, and Neil S. Greenspan, MD, PhD, senior editors of *Pathogens and Immunity*. Dr. Schinazi has invented numerous powerful antiviral agents including the antiretrovirals lamivudine (3TC) and emtricitabine (FTC) and was instrumental in the discovery of the anti-hepatitis C agent sofosbuvir. These drugs have saved millions of lives and, here, Dr. Schinazi shares in detail how these agents were invented, how they work, and how they are being applied. He also offers his thoughts about newer approaches to treat viral infections.

Dr. Schinazi is the Frances Winship Walters Professor of Pediatrics and Director of the Division of Laboratory of Biochemical Pharmacology at Emory University. He is the recipient of numerous awards and honors, including, very recently, an honorary doctorate from the Hebrew University Hadassah Medical School; his third honorary degree. Dr. Schinazi has had a formidable and truly remarkable career in the development of effective drugs to treat viral infection. An estimated 94% of patients worldwide who are being treated for HIV infection are receiving a drug that was invented by Dr. Schinazi. These agents have helped to save millions of lives through prevention and treatment of infection. Welcome to *Pathogens and Immunity* Ray.

## NEIL S. GREENSPAN, MD, PHD

How do you believe your family background in several countries and your experiences in those countries influenced your subsequent intellectual development?

## RAYMOND SCHINAZI, PHD, HON DSC

I was very fortunate to have both parents who were very pro-education. They suffered a lot themselves and were able to give the best education money could buy. But, it all started in Egypt where I was born—a country I love. When I was about 9 years old, my mother was very sick. She caught a yeast infection, and there was no medication in Egypt. Some of the third-world countries do not have access to medicines, unfortunately, even today. That was back in 1958-59, so you can imagine, trying to find a simple antifungal agent was almost impossible in Egypt. My grandfather was fortunate to know a pilot on TWA who brought some mycostatin from the United States, which is Fungizone for those of us who are more familiar with that name. It's available in every single lab in the United States for cell culture. That drug saved my mother, and I was very impressed. I thought, how fantastic that a simple drug like this can actually cure somebody. My mother went on to live to be 94 years old. That tells you that she had a long life, thanks to this drug. We were ready to bury her, frankly, and this small molecule was able to really destroy the yeast and make her whole again. That left a very big impression on me.

At that time, even at 9 years old, I spoke 3 languages fluently. I think languages are very important because they make you develop differently. Every language is like being a different person—knowing the culture of the country, speaking Arabic, speaking French, and speaking English. Later in life, I studied Spanish, because my parents lived in Spain. So again, I started learning a fourth language. I think, as you know, when you know a language, you can joke with people, you can understand the politics, you can understand their food, and so on. So, I think that has also helped my way of development, because it's not just learning about science, it's how to solve problems. And when you can solve problems with languages, understanding different cultures, that really helps.

## NG

I was going to follow up with what early influences steered you to science in general and antiviral pharmacology in particular, but you've certainly given one aspect of that answer already. Although that was a fungal infection that you experienced in your family—quite a powerful story—were there any other early influences that turned you toward science and your particular niche in science?

## RS

Yes, my uncle André Nahmias is a very well-known herpes virologist. He came to the United States in 1948. As a student, he couldn't get into medical school, so he went to Austin, Texas, and studied English literature, among other things. Then he went to study medicine at The George Washington University School of Medicine and became a pediatrician. He did his fellowship at Boston and then moved to Atlanta.

I knew about herpes viruses when I was 10 years old, thanks to my uncle. I wanted to be a bit like him. He was a big influence in my life. Eventually, I worked for him for a few years, and together we helped develop acyclovir with Burroughs Wellcome. So that was a great experience—with Larry Corey and André Nahmias. They published in *New England Journal of Medicine*, the first paper on the use of acyclovir for the treatment of herpes genitalis [1]. It's a fundamental paper. I was somewhat involved in this, and I wanted to learn more about how to conquer viruses, and that's where it started.

## NG

Do you have any strong interests apart from science?

## RS

I like fishing, in general—fly fishing and other fishing. I'm not good at golf. So, forget about that. And I try to, whenever I can, get on a boat. It makes me very relaxed to be on a boat.

## MICHAEL M. LEDERMAN, MD

There's an emphasis nowadays, in our schooling, on STEM—on having students focus their entire educational career on Science, Technology, Engineering, Mathematics. Do you think that's a good approach? Or do you think that a more balanced approach to secondary and college education helps in the development of a scientific career?

## RS

I think, when you are young, you have the opportunity to study many things, from history to science and mathematics and English and literature, and so on. Then, you have to specialize. I think it's important to become intense about science in order to excel in it, because there is so much competition right now. You don't necessarily have to know all 4 different STEMs. But focusing on, say, chemistry, biology, or engineering is great. I think having this plethora of opportunities is what you need today to succeed, but it's not essential because you will be normally exposed in life to other things.

## ML

After your postdoctoral fellowship, you moved to Emory. Why did you do that instead of joining pharma, which is where so many antimicrobial agents are developed?

## RS

After leaving England, I came to the United States, and I worked with Bill Pruso? at Yale, who was considered the grandfather of antiviral agents. He discovered idoxuridine, an antiviral compound used for the treatment of herpes keratitis.

And the winters in New Haven were not very favorable. They were very cold. So, I did apply to some of the major companies at one point. I remember asking Maurice Hilleman, may he rest in peace, who was one of the giants in vaccinology, if he had a position at Merck in the field of anti-virals. At that time, they weren't focused on antivirals, so there was no position for me there.

Pharma also wanted people who were chemists to stay in chemistry, biology to stay in biology, virology to stay in virology. They didn't intermix disciplines. I, on the contrary, was a chemist, who wanted to be a biologist, who wanted to become a virologist, who wanted to become a pharmacologist, who wanted to become a biochemist. And that's exactly what I did. I was a bit out of the box at the time, and I felt academia, for me, would be best. So, imagine, here's this young man coming from Yale to Emory as an organic chemist, and I'm actually swabbing the vaginas of women in a clinical lab. You learn a lot when you actually see the disease for yourself. When HIV came along, I was actually in a clinic. My lab was next door to HIV patients and you could see them outside the door dying slowly. That leaves a tremendous impact and motivates you to find a solution to the problem. Putting the clinical people and the PhDs side-by-side is so important.

## ML

And you point out an important value of being at a medical center where human beings are being cared for, and research is being done on ways to improve their lives. At pharma, you might be a little farther removed from that unless you have some intimate connection with a medical program. Is your approach to the development and invention of antiviral drugs distinguishable from the approach that pharma typically uses, or are methods similar but in a different environment?

## RS

I like rational design but everybody can do rational design. I am a bit more of a risk taker. I use my knowledge and intuition to develop drugs. Pharma is much more rational in its approach, and fortunately, that's not something I wanted to do.

## ML

It sounds as though you were in a position to take some risks that pharma, who are often risk averse and have a somewhat complex process in terms of the greenlight, might not be willing to.

## RS

Yes, absolutely. They rarely take risks, because they have to explain everything to management. It's what you find on the way to, sometimes. You find unexpected things. Pharma would never have discovered lamivudine (3TC) and emtricitabine (FTC), frankly. The whole story there is a good one. You have the idea of making oxathiolane nucleosides. What is an oxathiolane nucleoside? It is a nucleoside containing an oxygen and a sulfur attached to a carbon in a 5-membered ring. Now, any rational chemist, including pharma, will tell you that this is an impossible molecule to make; it will break down in seconds. They will give you 10 reasons why it will not be stable and why it will break down.

Well guess what? We made that. I actually went to my PhD mentor and showed him the structure in confidence, and asked, “Can we make that?” And he said, “No.” That was my former boss. That didn't stop us. We went to a meeting in Montreal, Québec, Canada—an International AIDS Society meeting. Dr. Bernard Belleau presented data on dioxolanes nucleosides—2 oxygen and a carbon. And I thought, “Wow, it's possible. They were all wrong.” I observed that and thought, if you can make 2 Os on one 5-membered ring, why not an O and S? I came back home, and we worked with some chemists and were able to make similar compounds but not yet lamivudine or FTC.

But there's also a bit of serendipity in science, as you know. So, we are trying to make FTC. In other words, hook up to the base a sugar with a 5-membered ring. And you do that with something called a Lewis acid. It turns out, the postdoc was a chemist from Korea. His name was Woo-Baeg Choi, and he didn't know anything about nucleoside chemistry, which actually helped. There was one bottle on the shelf in my lab containing stannic chloride, which is a Lewis acid. Nobody used stannic chloride then, but when he did, he got a 99% excess of the right enantiomers. There are 4 possible isomers: the alpha and the beta, and then the racemates — the left and right hand. And he was able to get exclusively 99% of the product was the beta derivatives — two of them. I can go into the chemistry and, of course, the rationale.

Tin(II) chloride binds to the oxygen—it has an affinity for oxygen, so it hooks up from the bottom of the ring and forces the base to come from the top. Instead of making all 4 isomers, you only make the ones with the top one. That shows you that good science with a bit of intuition, with a bit of luck, can bring something very special. And then the next problem was separating the two enantiomers. And it turns out that one enantiomer is less toxic than the other. Both are active. One is FTC and the other one is 3TC. And we actually licensed the process to, at the time, Glaxo, and they made 4 tons of lamivudine using our process. People don't know the story, but this is amazing. Four tons because of that luck. With all their chemists, with all their resources, they were unable to make what we did at Emory University with my friends Dennis Liotta and Woo-Baeg Choi.

## NG

That was a fantastic story.

## RS

There are stories to every one of my molecules. It's not an accident. It's a lot of hard work. In fact, one of the best stories is the story of the discovery of [the drug for] hepatitis C, and nobody knows it better than I do, despite other people claiming to have invented this molecule. There are basically two parts to the molecule. One is the drug itself, and the second part of course is the prodrug part. The prodrug part was well known; they used the McGuigan approach. But the story is a very good one because it involves some amazing people we hired at Pharmasset Inc., a company I founded. We had a vision. Very little money, but you don't need much money to make big discoveries, at least initially. And, we were very lucky at the time to be able to buy a real-time PCR machine, which at the time cost about a half a million dollars. So, technology helped us.

The other part was the discovery and development of replicon systems for hepatitis C by Ralf Bartenschlager and Charlie Rice. These [replicon systems] were critical. So having these two components, and the third component being people—qualified people who knew about virology. We knew about genetics of the virus, because we always assumed that the virus was going to mutate, and we'd have to counter that at some stage. We were able to find, in our library, a compound that worked against hepatitis C. It was actually a 2'-fluoro-cytidine, which has a 2'-fluoro group in the down position of the pentose ring. A very simple molecule. That compound was not potent, but it gave us a lead. And then we made a difluoro, which is gemcitabine. You probably know this drug that's used as an anti-cancer compound. Believe it or not, in cell culture, it has anti-HCV activity but is a very weak inhibitor of HCV in humans. And we never published that result. But we do have a patent filed somewhere. And then we made the 2'-fluoro-2' methyl. Now this is the genius. This is the new molecule that had never been made before. It wasn't easy to make. It's a lot of hard work. Nobody thought about making that at the time. Like nobody thought about making FTC, and I can go into why later if you wish. But clearly, this was a novel thing to put two groups at the 2' position: one of fluorine—and nobody would use fluorine in his right mind because we had the experience of FMAU and FEAU. Remember these compounds [that Jack Fox and Kyoichi Watanabe developed at Sloan Kettering] for hepatitis B that killed two patients. They licensed them to Lilly, and unfortunately the drugs killed some patients. So, nobody in his right mind would put fluoro in the 2' position. Well, we did. But we put it in the down position not the up position. There are two positions. So, we put also a methyl group at that position. And the compound was a cytidine analog, and the compound went all the way to the triphosphate, and the triphosphate inhibits the HCV polymerase. So, we were clapping and opening a bottle of champagne. We had the first major hit at the company. And we actually licensed that compound to Roche because we always need a big partner to develop these drugs.

But at the same time, I discovered that there was a weakness to the compound. The weakness was that the compound can get deaminated at the monophosphate level to the U analog. And we knew that from monkey work that the U analog is not active at all. It is totally inactive. But we also knew from a brilliant biochemist by the name of Murakami, a Japanese scientist who worked at Pharmasset. He worked out the mechanism of how this drug is metabolized in cells and found that the U analog, once it's formed, is actually phosphorylated. And the end product of this phosphorylation is the active ingredient in what we know today, as sofosbuvir. It has a long half life. That's why sofosbuvir can be given once a day. And it has a good affinity for the HCV polymerase; it's very selective for that. So, then the obvious thing to do next, was to make a prodrug of the U analog. We had a clue that it would be active also because we were never able to develop resistance to the cytosine analog. And the reason is quite simple, now that we know this. We actually produced two drugs, a C plus a U in cell culture. And it's almost impossible to develop resistant virus to what we call PSI-6130 since it formed two active nucleoside triphosphates. So, the next step, of course, was to make the prodrug of the U analog and that became sofosbuvir. We used the McGuigan approach because the actual derivative we used was off patent, so we didn't have to pay any royalties. We made the prodrug, and that became sofosbuvir.

Now, the data were presented at a meeting in Berlin by Ed Gane, professor of medicine at the University of Auckland, New Zealand. We went to New Zealand because they have a very accelerated development for investigational new drugs, especially in the area of hepatitis. And it's a long way to go, but it's worked out very well. He's a very well-known scientist now, thanks to this molecule. He gave the talk, and I couldn't even get into the room. It was packed.

And the data were shown that just on sofosbuvir alone, sofosbuvir plus interferon, we're getting sustained virologic response 12 and 24 [weeks] of 90+ %. I can tell you, we could hear a pin drop in that room. I was just outside; the door was open, and I could see what was happening. The audience was stunned.

At our board meeting for the company, we were obviously quite happy with the results because nobody had ever seen anything as potent and as effective as this. So, it was a wonderful time. And we called ourselves the Perfectovir group. “Perfectovir,” which is the perfect drug. Of course, you know, it's not perfect, because the power of this molecule is especially apparent when used with an NS5A inhibitor. And the NS5A inhibitor came later. Of course, Bristol Myers Squibb had already a very good one (Daclastavir). But unfortunately, they didn't come to an agreement. So, they had developed their own, there was a delay in developing the combination, but eventually we had a combination, which today we know as Harvoni, or Epclusa. These are fabulous drugs that work against every single genotype. And what is amazing is the need of this drug in a country like Egypt. Because 1 in 5 people in Egypt is infected with hepatitis C.

So, here's this young man who was kicked out by Nasser, from Egypt in 1962. And, in Hawaii, I got a call from the ambassador to Egypt asking me to come to Egypt, immediately. This was before the drug was approved. It was December 2011. In February 2012, I show up in Egypt for the first time in 47 years. The people greet me—all the military people, the same people who kicked my family and I out of Egypt. And they know I'm Jewish. Of course, they want our drug. They didn't know how to approach Gilead, and so on, but we found a way for Gilead to deliver the drug to Egypt for $1,000. They were ready to pay $3,000, by the way, but we negotiated it down to $1,000. So, I helped them. And then of course, they made their own generic formulation. They have great chemists there, they actually manufacture the drug in Egypt—in Alexandria, the city I was born in. They make sofosbuvir there. They formed a whole company, a whole structure, and now they deliver the drug for $60 a cure—a cure, not a treatment.

## NG

That's really amazing.

## RS

And, president El-Sisi, last year, invited me to Egypt for an ExCon meeting. He invited all the African leaders, all the African scientists to come to Egypt to discuss medical issues, but especially hepatitis C. And there, he offered the drug free of charge to every single African country, because he could make it very inexpensively. So, I raise my hat to him. Our own president, as you know, has just decided, earlier this year, to do the same thing, but it hasn't happened yet. There are more than 2 million people infected with hepatitis C in the United States. Egypt has come from being a red zone to where they have cured more than 5 million Egyptians of hepatitis C and rising every day. It was a lot of suffering, and every single family was affected in one way or another. And the first time I went there, as I told you, it was the military who invited me, because they had a lot of their soldiers dying of hepatitis C, and they wanted to do something about it. I don't think many people are able to say that one year, you go to Egypt to get an award, and then, the next year, you go to Israel and get another award. This time an honorary degree. So, I thought it was funny that I got involved in both countries. And I'm very pro-Egypt, and I'm very pro-peace. I want both of them to live peacefully forever, if that's possible. And I think peace through health is a very important concept that we need as scientists to push forward.

## ML

Well, Egypt should be proud of you, as we are in the United States for your amazing contributions to global health.

## NG

You've also thought about hepatitis B. Can you offer any thoughts about whether you think it's going to be possible to eradicate a virus like hepatitis B through your small molecule approach?

## RS

Let's put it this way, I think it's not going to be as easy as hepatitis C because there are two places where the virus can hide in the cell. One is the covalently closed circular DNA (cccDNA), which is a latent form of the virus and also, the integrated DNA, so you have to tackle both of them. And also, the general high level of replication. There are actually three problems. The replication, I think we can handle with the drugs we currently have. And for some of them, I was involved in the discovery of the compounds like telbivudine and lamivudine. FTC, unfortunately, is not being used for hepatitis B. But people do take it, especially when they take FTC for HIV, with tenofovir alafenamide. It does have an impact on hepatitis B, but nobody talks too much about that. But clearly, we have the replication of the virus, we have the cccDNA. I think you'll hear more about CAMs, or capsid assembly modulators.

A CAM is basically a compound that interferes with the capsid formation in one form or another. There are many mechanisms, it could be preventing the virus from getting into the nucleus by interfering with a capsid or actually deforming or displacing the genome inside the capsid. Or breaking it up. So, these compounds are well-known in literature. They've been dismissed by some people as being ineffective, but I believe they're wrong and will be proven to be wrong.

We have now developed some quite remarkable CAMs. Data have been presented at the EASL meeting, and more will be presented later this year AASLD, in Boston. Clearly, these CAMs give you about as much as [a] 6 log drop in virus. Even a dose as low as 10 milligrams per day in a human being, that's pretty impressive. None of the other compounds can do that. The CAMs that are out there, these are capsid effectors, are super potent, femtomolar, well, I will say picomolar in culture, but they are very active in humans. But, most important, they not only bring the virus load down, they also have an impact on the HBs antigen. And that is directly linked to hepatitis B integrated in the genome of humans. So, the more HBs antigen you have, the worse it is. So, they're beginning to see a decline, and the decline is still going on even after 40-plus weeks of treatment. And the slopes are still going down. Unfortunately, the patient starts at 4 logs of HBs antigen, not two logs. If it were at 2 logs, we would have probably reduced it to undetectable. But it will take a lot more time. We estimate it will take anywhere from 1.5 years to 2 years when you have that much HBs antigen to start. Remember, with hepatitis B, you also have the immune system that's intact. So, you can basically tackle both: The immune system is working in your favor, unless of course you're immunocompromised. Plus, the drugs that we have. And we usually use something like entecavir plus the new CAMs. One of them is called ALG-184. That's the CAM I'm talking about. That's the one that gives you a tremendous response.

Everybody wants a home run with hepatitis B just because we had the home run with hepatitis C. They thought it was easy. It's not easy. It was not easy at all. But they seem to think “Well, you know, we cured hepatitis C; well, we can do the same thing for HIV. We can do the same thing for hepatitis B,” but that's not the case. But, going back to hepatitis B, I think, if we continue treatment for, say, at least 1.5 years to 2 years, we'll have the HBs antigen to undetectable, at least, with the current tools we have. The hope is that the patient will seroconvert at that point and once they seroconvert, you have a cure.

## ML

When you give these CAMs, can you provide some estimate as to the turnover of infected cells?

## RS

Yes, they used to think the turnover of infected cells was very long, but people have done some work, and they think the turnover is about 2 weeks. The problem is that you need 2 to 4 weeks, let's say. But even with that, the problem is you got to make sure that the drug is constantly there, both the nucleoside or whatever else you plan to use. It could be an immunological approach too, I'm not against that either, or siRNA approach, or some other approach, plus the CAMs. But the CAMs are going to be the driving force, a bit like what you have today with the nucleosides. The nucleosides are the driving force (the cornerstone), they give you 2 log plus for HIV, and then you use, of course, an integrase inhibitor or a capsid inhibitor with HIV.

The new HIV capsid inhibitor looked very promising. Lenacapavir, specifically. So I think it's going to be practically the same thing for hepatitis B. Now, I'm not saying that it's going to be a home run or a slam dunk. I'd be very, very surprised. I think we'll get initial cure rates of maybe 30% to 50% with this material. And that's going to be incredible. Because remember, there are at least 250 million people infected with hepatitis B. So, I really think that the opportunity is there, and I think we'll fine-tune and improve things with time. We'll find what works best. Because if you can find 2 very powerful drugs that you can put together—I don't think we have anything like the CAMs today — so if you combine a CAM with something else that really works well, you may be able to reduce the cure time to say, 6 months or 1 year. And that's what we're looking for down the road. But with the current status, the drugs that we have today plus the CAMs I think it will take 1.5 to 2 years to clear the HBs antigen. And once you have that, you will seroconvert. Remember, also there is spontaneous seroconversion; there are people [achieving] it with interferon because we know with interferon treatment for 1 year, you get 5% cure rate, approximately.

## ML

In natural infection, some people are cured spontaneously.

## RS

Correct. So, you will get a small percentage. You don't really need a control group for these studies, in my opinion. You can do a control group if you want. But, I think we all know when you stop therapy, invariably, the virus comes back. So, to me, a perfect cure would be you stop the therapy—forget about all the markers, it's helpful to have all these markers, or surrogate markers, if they are surrogate, I don't know if they're useful yet.

But we're going to learn a lot about the markers that are effective, and prediction, and who can be cured fast, who can be cured slowly, who cannot be cured at all. And we'll need some other methods. So, I think a lot of lessons are going to be learned in the next year or two in terms of hepatitis B. Once we can cure some patients reliably, like 20%, or 30%, or 40%. But again, as I said, everybody's looking for the home run; that's not going to happen. Let's be cautious here. It's a much more difficult virus to treat than hepatitis C.

## ML

So, you're optimistic about having some impact on curing hepatitis B. Do you think that we'll be able to have—and what are your thoughts about the directions to get — a scalable HIV cure strategy?

## RS

Well, a “cure” depends on how you define it, but I think a functional cure is definitely possible for HIV. But to completely eliminate the virus where the virus doesn't come back when you stop whatever therapy you have, that's going to be very, very hard. It's like trying to find a cure for herpes viruses, it's going to be impossible.

Herpesvirus integrates into the nerve cells so it's difficult to reach the virus. People have thought about using horseradish peroxidase and other things that [undergo] retrograde axonal transport, but nobody has been successful. The only thing that cures herpes, by the way is age. So, the older you get, the less likely you are to have a recurrence. So that's the only advantage of getting old, I guess. So, I think it's going to be much more difficult. However, I am optimistic. I hope somebody finds something, but I don't think it's going to be by traditional approaches. We probably don't have the answer yet, because we don't quite understand all the reservoirs. We don't even understand where the virus hides. That's the biggest problem. Everybody is shooting blindly in a barrel. So, I think it's going to be very difficult. Not impossible, but you have to be optimistic.

Now in terms of functional cure, I strongly believe it's possible. We already have, in a way, a functional cure with the Truvada and Descovy, and these types of drugs. It's a functional cure, whether you like it or not. You give the drug forever, you never get recurrences, and you're safe. Of course, you have to take the pills every day. Now, there are ways to improve on that, and people are working on that, in terms of long-acting delivery. But I think there are other ways of doing it. One possibility, I think, is with antibodies. I think antibodies are underrated today by a lot of people. In particular, the drug companies. They will eventually start working on antibodies, Antibodies are going to be very, very useful, provided you don't get antibodies to the antibodies. That's the biggest problem, as you know, the biggest challenge.

## ML

Useful in terms of their activity and duration of their activity *in vivo*, or useful in terms of providing a mechanism for eradication that's not provided by the current agents?

## RS

Well, it's not eradication. You're suppressing it. The virus is still there, but you're suppressing it very effectively. And we've seen that with monkey experiments where in young monkeys as well as adult monkeys, you can suppress the virus for a very long time. I'm talking about years. You can do it with Truvada. But the problem is, in some cases with adult monkeys, it's actually only a small percentage of monkeys produce antibodies with sustainable levels. And that's the problem. And the reason is that you have antibodies formed against the antibody. If you can reduce that or eliminate that, I think we have a winner. It would basically take over the whole market, in my opinion, of the current drugs that we have. You can use it for a therapeutic in people who are already infected, as well as for prevention. So, it would be really wonderful from that perspective. And if you put it, let's say, in an Adeno-associated virus (AAV) vector, and you put the right cocktail of antibodies with the right conditions, you have an amazing opportunity. So that's something that I support fully, and I am getting more interested in destroying my antiviral legacy with antibodies.

## ML

So, what's your favorite vector for getting cellular production of antibodies?

## RS

AAV. There are many types of AAV that you can have that target the liver; it's a one-time intramuscular dose, and that's it. And there has been evidence in animal models that these antibodies can persist a long time. A lot of work has been done in mice as well as in monkeys. I'd love to see that in humans, and I think there are people starting to do studies along those lines. So, I'm very excited about that. I think CRISPR technology may be a bit of science fiction. I'm not against that, but where are you going to go? You still have to deliver. So, I think there are a lot of hurdles, but I encourage people to work on it.

## NG

It's so refreshing to hear someone such as yourself give a serious discussion of the challenges of cure, as opposed to just throwing the word around loosely, as if it's inevitable that we're going to cure every disease. As you said, hepatitis C is an amazing achievement, but it's its only possible because of certain features of the virus and its replication cycle and so forth. And, other viruses, other infectious agents present different kinds of challenges. And, prior to the hepatitis C drugs, the only cures that were routine that I encountered were antibiotics for bacterial infections. There are very few chronic diseases that we cure. So that sets up my next question, which is what about curative, small molecule drugs or other approaches for other more common viruses that are not chronic viruses?

## RS

Well, I think the traditional discovery of small molecules for yellow fever, Nipah virus, etc., is going to continue. The big question is: Is big pharma interested in this? Because, practically, there is not much money to be made. And, how do you get the industry to engage? I've seen Janssen do some wonderful work in Africa with TB, for example, and also their new drug on dengue that was discovered with our colleagues at University of Leuven in Belgium. They've done an amazing job. But is that going to see the light of day? So, I think this is where NIH, BARDA, WHO and other organizations, even small countries where these viruses are endemic, could play a big role, with the help of American scientists who are fairly advanced in this area, European and Chinese scientists and everybody else—they need to get together and set money aside for the development of these molecules, so we're ready when these viruses cross our border. With global warming, already West Nile is a big problem in the United States. People don't talk about it, but there are over 1 million people getting infected.

## NG

Can you crystallize any unusual insights that you've had that have helped inform your career and lead to the successes you've had? You've hinted at several, in terms of your comparison between your ability to be more free ranging in an academic environment versus in a typical pharma environment.

## RS

It's not just me. As you know, these molecules are not invented by one person. There are a lot of people involved. The key is quality people. We have to train more virologists, we have to educate them about drug discovery, drug development, we have to teach them about small molecules, we have teach them about big molecules, we have to teach about oligonucleotides, and mechanisms. There is a lot of teaching to be done. And working in a lab like ours, young people can learn a lot. But it's very hard nowadays to find talented virologists, as you probably know. We need to have a program to bring in more virologists to become interested not only in drugs, but also vaccines.

## ML

You've developed a lot of very successful molecules. What's your favorite antiviral that never made it and why?

## RS

I can think of two drugs immediately for HIV that I thought were going to be blockbusters. One was amdoxovir. You may be familiar with that molecule. It's a dioxolane purine nucleoside that went all the way to phase two clinical trials. It was being developed by Triangle Pharmaceutical. Triangle got sold to Gilead. It sat on the shelf at Gilead for a year. And then, after a lot of discussion, it returned to Emory University, and we tried to rekindle it. We spent millions of dollars developing it here in Atlanta, with a small company that I had formed. We even had the clinical studies ready in Argentina. But unfortunately, we couldn't recruit enough patients, because we had all these other wonderful drugs available. And we just had to throw in the towel. It was very sad, because I knew that drug was as good as tenofovir, with less problems and more potency than tenofovir. And it could have been a great addition to our armamentarium for HIV.

The other one was a drug that we licensed to Incyte, believe it or not, at a time when they were getting away from the gene sequencing activities, before the Jak inhibitors came along. This compound was probably the most potent nucleoside at the time on the planet. We had done a pharmacokinetic (PK) study with infected patients. And after one single dose, we had almost .5 log drop. It was actually interesting. Most people don't test drugs after one day, but I did. A lot of people are doing it now, but at the time, it was totally novel. This may be a creative way of thinking, but why not? If it's got to be so powerful that we know the virus load comes down very fast. And we showed a reliable .5 log drop in all the patients. It was a PK study in infected persons, but we had saved the plasma, and we were able to measure the viral load drop. We actually published that paper [2]. And as I said, a lot of companies have copied us now, because you don't need to do long-term studies. You can find out very quickly whether the compound is going to work in the clinic or not, even after a single day. So, that compound was so powerful, and I think it was completely mishandled. The combination they selected was not the right one. They combined some of the older drugs with our nucleoside. Eventually, we found out the company was not really interested in antivirals. They went on to the Jak inhibitors. But, I still think it's a wonderful compound. But today, we have a lot of very good drugs for HIV.

## ML

Jak inhibitors have demonstrated efficacy in a number of settings, do you think there are other or any host-targeted approaches for prevention of HIV infection that will turn out to be useful? You're optimistic about introducing the production of antibodies *in vivo*. But are there agents that you think will target host elements that the virus needs for propagation?

## RS

A lot of people are working in this area. And we're discovering new host factors that are important for HIV and other viruses. Recently, we saw some papers on sphingolipids and enzymatic mechanisms that are involved in lipid metabolism, and so on. So, I think they're not targeted specifically at the virus. I think it's great. There are also people who are in the contraception area that are looking for compounds they could use in combination with the concurrent contraception devices that we have to slowly release to prevent STDs, including HIV, and they're looking for polyanion compounds that could be effective, algae-type of extracts, and so on, that are charged molecules that could affect the receptor to the virus. So, I'm all in favor of that, but my personal career has been focused on mechanism, mechanism, mechanism. So that's what we try to find: What is the mechanism, and confirming the mechanism. Obviously, to me, primarily, the mechanism, in the case of HIV, has been the polymerase. It also has been polymerase for hepatitis B, HCV, HPV, but we also look at other targets that could be useful, like the CAMs I mentioned earlier.

## NG

Have you thought at all about mRNA-based therapies?

## RS

I think that's an evolving field and very exciting. We've seen the success of the COVID vaccine with mRNA. They're not perfect vaccines, but they certainly did their job as best as they could. They are not durable, unfortunately, so I think there is room for a lot of improvements. But again, they went remarkably fast, it has been very successful. It's a proof of principle that they work, and now we can apply them to a lot of exotic viruses, a lot of exotic diseases, and even not-so-rare diseases as well. It's an evolving area, and the numerous companies that have now started up all over the world, especially in the US—even one in Atlanta, believe it or not, that is making these mRNA vaccines. They have to be tested, of course, in animals to demonstrate that they work both therapeutically as well as for prevention. And they could be extremely, extremely good. So, I'm all in favor of that. I think if we better understand the requirements needed in terms of expressing the full length of RNA or what are the important parts of the RNA that are essential for its effectiveness and durability, that could be very, very good.

## NG

I was thinking not only vaccines, but also actually encoding other sorts of proteins that inside the cell can interrupt cellular processes necessary for the virus, whether they're host or virus encoded.

## RS

I think we're going to see a lot more of that at meetings. It's going to be very, very exciting. And it's certainly a new area, a refreshing area, because we need some changes. We can't keep doing the same thing forever.

## ML

If a physician is serious about a research career, what do you say about the need for, or recommendations for formal training in science, getting a PhD?

## RS

If they don't want to do a PhD, that's fine with me. I think the key is having protected time to do research. That's the biggest problem. I've been in this business for more than 45 years. We have MDs embedded in our group. They don't necessarily have PhDs. But they behave like PhDs, and they're also surrounded by PhDs, which will help them, and I think that's the goal. When you get an MD-PhD student in your lab, you feel honored that they chose your lab. You know that these people are motivated. And you know that they are going to spend more time to get their degrees. And they will work very, very hard for you and for themselves as well. But I've seen MDs excelling in our labs in the past and going on to do big things.

## ML

I have just two more questions for you. Who are your favorite teams in Premier League and Serie A?

## RS

Well, I like I like Liverpool. You know why.

## ML

No, you must tell me why you like Liverpool.

## RS

Mohamed Salah. Egyptian. He is amazing. Liverpool has come from nowhere. And I think it's a wonderful story. An African coming to England and being successful. In fact, a lot of Africans have done an amazing job, Mbappe in France, and it's remarkable the evolution that's taking place in soccer, football. I'm happy to see that happening. It's a lot of fun. And it's great to see these young men playing beautifully. And with such talent and speed. I wish we could have him on my team discovering drugs for HIV. He's a fast runner. Very fast runner.

## ML

You bet. Well Ray, thank you so much. It's been a thrill for me and I'm sure for Neil to have you talk to us for *Pathogens and Immunity*.

## Figures and Tables

**Figure:**
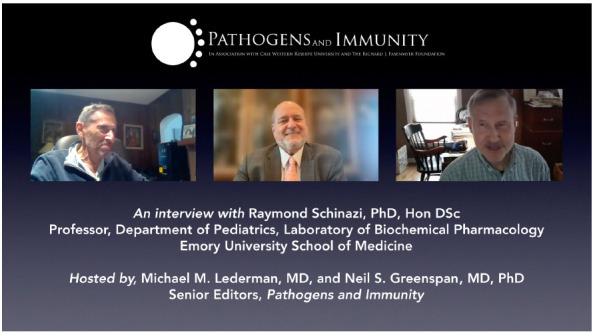


## SUPPLEMENTARY DATA

Supplementary materials are available at the *Pathogens and Immunity* website. Supplementary data may be provided by the authors to benefit the reader. Supplementary data are not copyedited and are the sole responsibility of the authors. Questions or comments related to supplementary materials should be addressed to the corresponding author.
